# Should Core Needle Lymph Node Biopsy be a Relevant Alternative to Surgical Excisional Biopsy in Diagnostic Work Up of Lymphomas?

**DOI:** 10.5152/eurasianjmed.2023.0060

**Published:** 2023-06-01

**Authors:** Yaşa Gül Mutlu, Berrin Balık Aydın, Aslı Çakır, Özlem Canöz, Cengiz Erol, Ömür Gökmen Sevindik

**Affiliations:** 1 Department of Hematology, Istanbul Medipol University, Istanbul, Turkey; 2 Department of Pathology, Istanbul Medipol University, Istanbul, Turkey; 3 Department of Pathology, Erciyes University, Kayseri, Turkey; 4 Department of Radiology, Istanbul Medipol University, Istanbul, Turkey

**Keywords:** Core needle biopsy, diagnostic, excisional biopsy, interventional, lymphoma

## Abstract

**Objective:**

Surgical excisional biopsy is accepted as the standard of care approach in the diagnosis of lymphomas. Financial issues related to the increased cost and the invasive nature of the procedure forced physicians to use some alternative diagnostic methods. Percutaneous core needle biopsy, which gained a reputation for the diagnosis of lymphomas with the advent of improved pathological, immunohistochemical, and molecular analysis, made it possible to have an accurate diagnosis with limited tissue samples. In this retrospective study, we aimed to compare the diagnostic yield of surgical excisional biopsy and core needle biopsy.

**Materials and Methods:**

This study included 131 patients who were diagnosed with lymphoma with a nodal biopsy which was acquired via surgical excisional biopsy or core needle biopsy between 2014 and 2020 in our center. Around 68 patients underwent surgical excisional biopsy and the remaining 63 underwent core needle biopsy. Samples that allowed to the identification of the exact tumor type and/or subtype were accepted as fully diagnostic. Sufficient amount of tissue that the pathologist could have any suspicious findings considering malignant lymphoma was classified as partial diagnostic group. Inadequate samples were the ones who were not enough to report any final diagnosis.

**Results:**

The patients who underwent a core needle biopsy were significantly older than the patients who underwent to surgical excisional biopsy (56.8 vs. 47.6, *P* = .003). Despite the full diagnostic ability of surgical excisional biopsy outperformed core needle biopsy (95.2 % vs. 83.8 %, *P* = .035), in 92.6% of the patients whose tissue samples were obtained via core needle biopsy were accepted to have a sufficient diagnosis to initiate the treatment and not required a second biopsy, which was comparable with the ones achieved by surgical excisional biopsy (92.6% vs. 95.2%, *P* = .720).

**Conclusion:**

According to the results obtained in our study, we may conclude that core needle biopsy is a viable and comparable alternative to surgical excisional biopsy, offering a less invasive and less-expansive approach.

**Main Points**

- Core needle biopsy (CNB) alone has a highly satisfying diagnostic ability according to the results of the study.
- Comparison of total diagnostic yield which allowed the patients to proceed with appropriate treatment was statistically not different between CNB and surgical excisional biopsy group.
- Core needle lymph node biopsy is an alternative diagnostic approach in workup of lymphomas, not only in patients who have deep and un-accessible lymph nodes but also the ones who have superficial lymph nodes.

## Introduction

Lymphoid malignancies are the tumors of immune system originating from B and T lymphocytes and rarely natural killer (NK) cells. Lymphomas generate an extremely heterogeneous group of disorders based on their biological, molecular, and genetic features, histological forms, sites of clinical presentation, and response to treatment. This heterogenetic complexity led to the development of some controversial and complicated classification systems. The World Health Organization (WHO) classification for lymphoid neoplasms is classification that is accepted all around the world and was recently updated in 2016.^1^

Diagnosis of lymphoproliferative disorders is based on obtaining a tissue sample and histological examination of this sample by hemato-pathologists. Aside from other malignancies, lymphoma diagnosis needs much more sample to observe whole architecture, especially in lymph nodes. Diagnostic methods can differ according to the preference of the center, logistic, and financial factors. Surgical excision biopsy (SEB), percutaneous fine-needle aspiration (FNA), and core needle biopsy (CNB) are the main sampling modalities in this regard. 

Fine-needle aspiration is a faster and cheaper technique when compared to the CNB and SEB. However, due to its high false-negative rate and low specificity, it is not accepted as a proper choice for the evaluation of lymphadenopathies for hematological malignancies.^2^ The WHO system for lymphoma classification relies on histological findings from excisional biopsies.^3^ Also, current lymphoma guidelines like the European Society for Medical Oncology (ESMO) and National Comprehensive Cancer Network emphasize SEB as a standard of care for the diagnosis and classification of lymphoid neoplasms.^1,4,5^

The excisional biopsy of a whole lymph node is regarded as the gold standard for the diagnostic workup of lymphomas; however, CNB should also be a viable alternative to this approach. European Society for Medical Oncology guidelines indicate CNB as an alternative diagnostic approach in lymph nodes which are not easily accessible.^6^ Before the advent of recent modern immune-histological methods, pathologists generally needed to investigate whole background architecture of an excisional biopsied tissue to make certain diagnosis and sub-typing of a lymphoma. But nowadays, they can make a final diagnosis and sub-typing with small tissue samples. Even SEB is accepted as the gold standard approach in the diagnosis of lymphomas, CNB is increasing in frequency and gaining more reputation.^7^

Another issue is that surgical excision of a lymph node or a tissue requires sedation or a general anesthesia, which makes it a more invasive and costly approach throughout the process of diagnostic workup. However, CNB can be applied by local anesthesia and can be finalized in less than half an hour. Also, it is much cheaper when compared to SEB. One other major advantage of CNB is the remaining tissue which allows a more accurate follow-up after the initial treatment.^8^ Core needle biopsy offers a less-invasive and cost-effective approach and possibly serves as a perfect alternative to excisional biopsy.

With these regards, we wanted to compare the diagnostic yield of CNB samples comparing with the samples obtained via excisional biopsy from our center. 

## Materials and Methods

Biopsies performed with CNB or SEB technique to rule out malignant or non-malignant pathologies were retrospectively retrieved from the archives of the department of pathology at our institution from the year 2014 until 2020. All of the CNB and SEB biopsies in the archives of pathology department were evaluated regardless of the samples’ origin of department. All specimens were evaluated at a tertiary University Hospital by dedicated hemato-pathologists who were experienced in the diagnosis of lymphoproliferative disorders.

**Figure 1.**
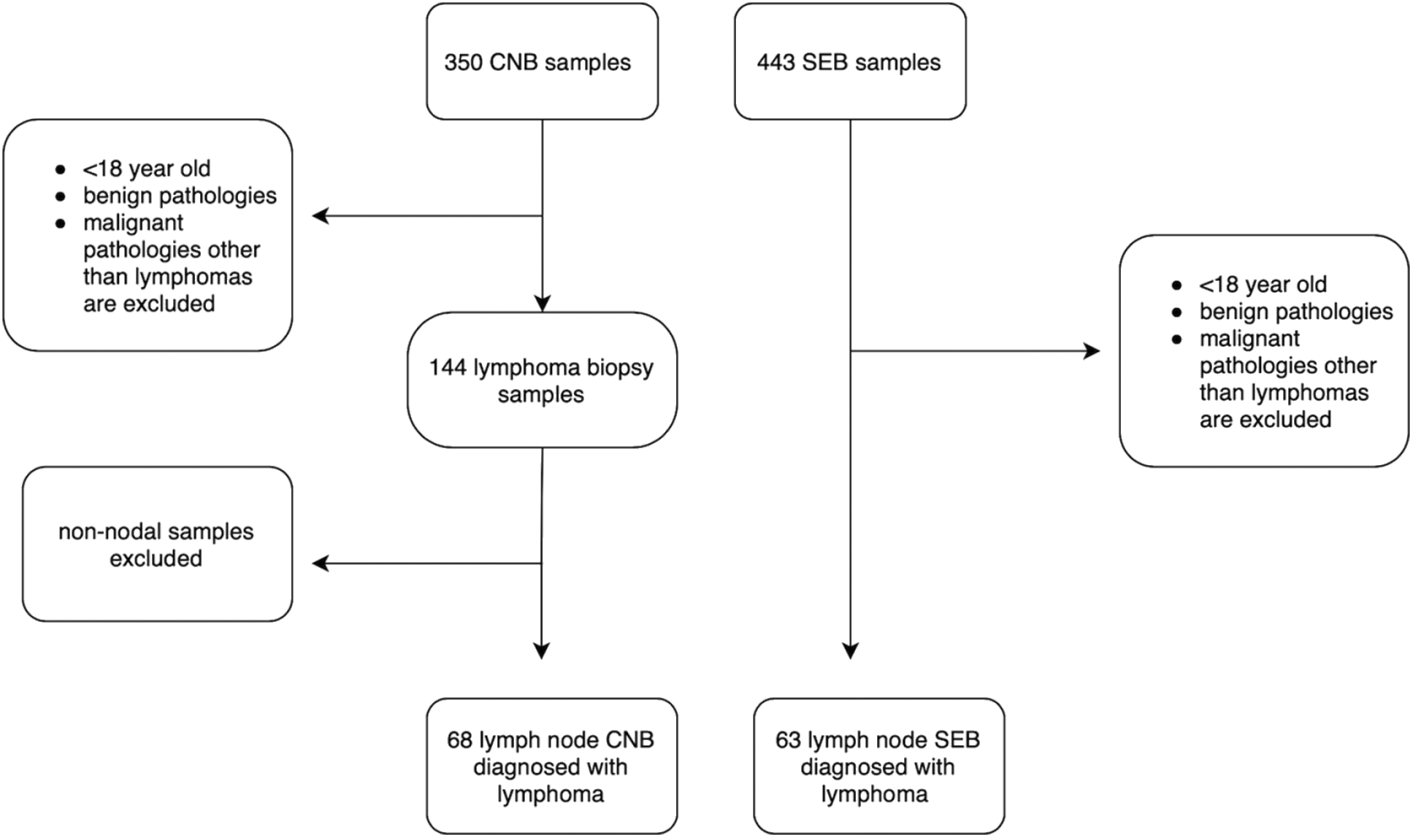
Flow chart defining the inclusion, exclusion criteria, and the production of final dataset.

Patients who were under the age of 18 at the time of biopsy and biopsies which revealed a benign pathology and the biopsies which revealed a malignant pathology except lymphomas were excluded. Totally, 753 biopsy samples were evaluated. The total number of samples obtained with SEB was 443 and CNB was 350. After excluding the patients who were under the age of 18 at the time of biopsy, benign pathologies, non-lymphoma malign pathologies, and non-nodal samples, we had 68 biopsies to analyze for CNB group and 63 biopsies for SEB group (Figure 1). A written informed consent was obtained from every patient who was included in the study. 

Samples were separated into 2 groups consisting of SEB and CNB. All demographic data including age, gender, patient, and disease-specific characteristics and the location of the specimen obtained were recorded alongside the final diagnosis. All the biopsies were re-classified into 3 groups according to the sufficiency of the diagnostic yield “fully diagnostic,” “partial diagnostic,” and “inadequate.” Samples that allowed to the identification of the exact tumor type and/or subtype or which were accepted to be sufficient for the initiation of the lymphoma treatment were grouped as fully diagnostic. Sufficient tissue that pathologist could have any suspicious findings considering malignant lymphoma classified as partial diagnostic group. The last group was inadequate tissue group as its name describes not enough sample to conclude any diagnosis, generally, these samples were necrotic or quite small samples. Also, all CNB samples were evaluated as if there were any need for an additional SEB to make the final diagnosis or a diagnosis to initiate the proper treatment. 

Samples were classified according to the dimensions of tissues, smaller than 3 cm, between 3 and 6 cm, and larger than 6 cm. F-fluorodeoxyglucose (FDG) avidity of the lymph node was recorded, if the patient had a Positron Emission Tomography (PET) scan before the biopsy. Imaging method before the biopsies, sample number of CNB tissues, and size of the core needle were also recorded. 

All CNB were performed by 1 interventional radiologist using coaxial biopsy technique. After local anesthesia, 17-gauge coaxial needle (Argon Medical Devices, Inc. Athens, Tex, USA) was inserted into the lymph node under ultrasound (US) guidance in superficial locations and under computed tomography (CT) guidance in deep locations. An 18-gauge core biopsy needle (Tru-Core II Biopsy Instrument, Argon Medical Devices, Inc) which has 2 cm tissue core length was then introduced through the coaxial needle to reach the lymph node and fired at least 8 times to obtain adequate biopsy samples from different regions of the lymph node. Core needle biopsy samples were just visually inspected by a dedicated interventional radiologist and there was not any rational sample competency assessment at the time of procedure. 

This study was approved by institutional local ethical committee with the approval id of E-10840098-772.02-793.

### Statistical Analysis

Variable distributions were assessed by the Shapiro–Wilk normality test. According to the variable distribution, Student's *t*-test or Mann–Whitney *U* test was applied for the comparison of groups regarding quantitative data. Categorical variables were compared by the *χ* ^2^ test and fisher’s exact test accordingly. Statistical analysis was performed using Statistical Package of Social Science (SPSS Inc., Chicago, Ill, USA), version 22.0 for Windows. Data were expressed as median (range) and a *P* value less than .05 was accepted as statistically significant. 

## Results

Totally, 131 lymph node biopsies were evaluated retrospectively according to the pre-defined inclusion and exclusion criteria. Patients’ cohort consisted of 78 males and 53 females, and the median age was 55. A total of 68 (51.9%) of 131 histopathologic samples were CNB and 63 (48.1%) were SEB. There was a statistically significant difference between age in 2 groups. Median age was 56.81 in CNB group and 47.57 in SEB group (*P* = .003). When we checked out the biopsy locations, most of the samples were obtained from head and neck in both groups (44.1% and 65.1%). Patients’ demographics and biopsy locations are summarized in Table 1. When the locations of biopsies obtained were compared between SEB and CNB groups, there was a statistically significant difference, and this difference was attributed to an increased number of biopsies from head and neck region in favor of SEB (65.1%) and vice versa from abdomen in favor of CNB (44.1%). Samples that were obtained from deep lymph node stations such as paraaortic, mesenteric, splenic, iliac, or mediastinal were favored in CNB group with the rate of 30.9%, comparing to 4.8% in SEB group (*P* < .001). The median number of samples that were obtained with coaxial technique in CNB group was 11.5 (2-45). There was not any significant difference between number of samples and diagnostic ability in CNB group. 

The lymphoma frequencies and sub-types in both groups are also presented in Table 1. The most common lymphoma subtype in both groups was Hodgkin lymphoma in terms of diagnosis (CNB 27.9% and SEB 41.3%). The distribution of non-Hodgkin lymphomas was different in the 2 groups. While Diffuse Large B Cell Lymphoma (DLBCL) (16.2%) was the most common lymphoma subtype in CNB group, T-cell lymphoma was the most common in the SEB group with a rate of 15.9%. Follicular lymphoma was the most common indolent lymphoma type in both groups (CNB 14.7% vs. SEB 14.3%). There was a limited number of patients in both groups which were categorized as atypical lymphoid proliferation, 4.4% in CNB group and 1.6% in SEB group, respectively. 

PET-computed tomography was the most commonly used imaging technique in patients with lymphadenopathy. Around 36.6% of the patients had a PET-CT before the biopsy. Other imaging techniques which were used less commonly were ultrasonography (19.1%), CT (9.9%), and magnetic resonance imaging (2.3%). 32.8% of the lymph nodes in diagnostic sampling were smaller than 3 cm in size, 28.2% were between 3 and 6 cm, and 2.3% were larger than 6 cm in size.

**Figure 2.**
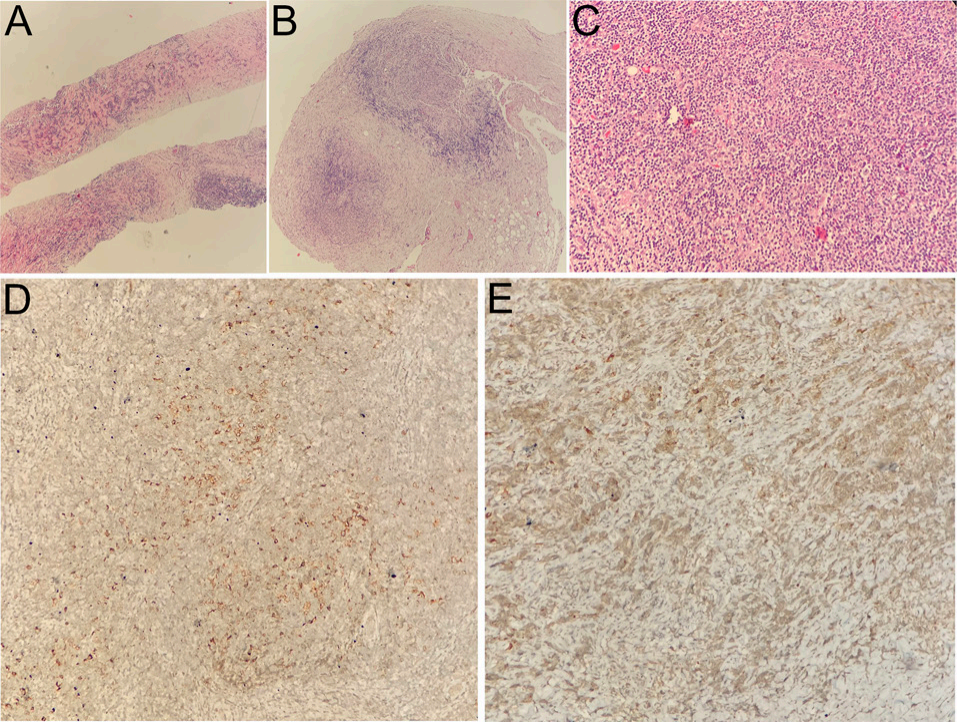
(A) Core needle biopsy of anterior mediastinal mass reveals sclerotic areas and small lymphocytes (HE, ×40). Diagnostic result could not be obtained in the immunohistochemical study applied to very few atypical suspicious cells. Incisional biopsy showed both areas similar to core biopsy (B) (HE, ×40) and areas rich in atypical cells (C) (HE, ×100). (D) CD30 expression in foci rich in atypical cells (CD30, ×100) and (E)CD30 expression in sclerotic foci (CD30, ×100) in incisional biopsy. This case was diagnosed as atypical lymphoid proliferation in core biopsy and classical Hodgkin lymphoma in incisional biopsy.

**Figure 3.**
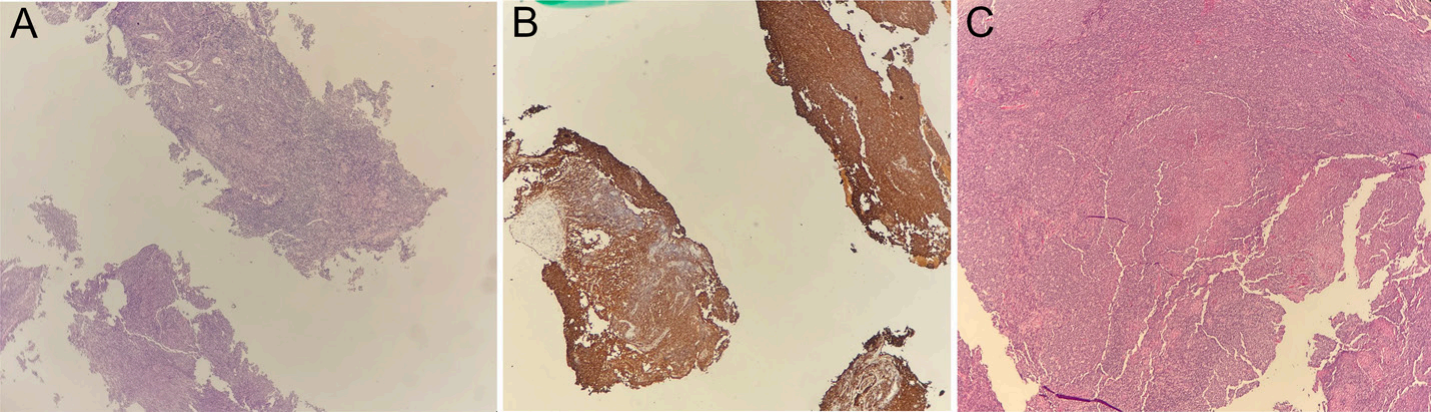
(A) In the core biopsies taken from the submandibular region, a lymphoid tissue sample consisting of partially monotonous cells and partial involvement is observed (HE, ×40). (B) Cores with diffuse and patchy staining are seen with CD20 (CD20, ×40). Biopsy with other immunohistochemical markers (bcl6, CD10) was reported as B-cell lymphoma of follicle center origin, but excisional biopsy was requested for subtyping and grading. (C) Grade 2 follicular lymphoma with partial lymph node involvement was diagnosed in excisional biopsy (HE, ×40).

When the diagnostic ability of the 2 techniques was evaluated, inadequate samples were 2.9% in CNB group and 3.2% in SEB group (*P* = .939). 89.3% was accepted fully diagnostic with regard to all samples. Fully diagnostic ability was 83.8% in CNB and 92.6% in SEB group (*P* = .035). Totally 66 (97%) of patients in the CNB group and 60 (95.2%) of patients in the SEB group either had a fully or partial diagnostic ability (*P* = .938) (Table 2). Only 5 (7.4%) patients who were in CNB group needed an excisional biopsy and 3 (4.8%) of the patients in the SEB group needed a second biopsy. Two of the patients who needed a second biopsy after CNB were diagnosed with Hodgkin lymphoma and the others were diagnosed with non-Hodgkin lymphoma. Total diagnostic yield which allowed the patients to proceed with appropriate treatment was 92.6% and 95.2% for CNB and SEB groups, respectively (*P* = .720) (Table 3). Two samples from different regions are detailed in the Figures 2 and 3. 

Hodgkin lymphoma was the most common lymphoma subtype in both CNB and SEB groups. Diagnostic yield of the CNB for Hodgkin lymphoma was 90.5% and 100% in SEB group (*P* = .194). Regarding all non-Hodgkin lymphoma sub-types, the diagnostic yield of CNB and SEB was 82.6% and 94.4%, respectively (*P* = .173). The diagnostic yield of CNB and SEB among patients who were diagnosed with DLBCL was 100% for both, and regarding the major indolent lymphoma sub-group, patients who were diagnosed with follicular lymphoma, the diagnostic yield of CNB was 80% and SEB was 100% (*P* = .474).

## Discussion

The major advances in the field of hemato-pathology, especially the novel immune-histochemical studies, made it possible to obtain a diagnosis of lymphoma even with small-sized tissue samples. The growing confidence and ability of interventional radiologists who apply CNB in a regular fashion have also appealed to the interest of clinicians to refer their patients to a less-invasive alternative. This gradually increasing confidence in CNB has also led to studies in which the diagnostic ability of CNB was evaluated.^9,10^ A meta-analysis of Frederiksen et al^3^ has indicated a 74% diagnostic yield of FNA/CNB in 5707 nodal and extra-nodal samples obtained between 1989 and 2012. Another review by Seviar et al^11^ has documented a 79%-97% diagnostic yield of CNB as the first diagnostic tool, when combining results of 13 different trials which were held between 2015 and 2020. This review has reported a median diagnostic yield of 91.7%. In the comparison group which included samples obtained by SEB, the diagnostic yield was reported between 93.5% and 100% with a median of 97.5%. We have observed an 83.8% fully diagnostic ability of CNB and 95.2% of SEB (*P* = .003) according to the results obtained in our study. Regarding fully diagnostic ability, there was a statistically significant difference between 2 groups. But when the diagnostic yield was the concern, only 5 out of 68 patients who underwent CNB required a re-biopsy with SEB in order to be treated according to a proper diagnosis. Core needle biopsy, itself, allowed 92.6% of patients to proceed with the appropriate treatment accordingly without a need for a re-biopsy, and this ratio was 95.2% in SEB group (*P* = .720). 

In routine clinical practice, CNB is much more preferred to evaluate lymph nodes that are not easily accessible or in patients who are prone to complications of anesthesia regarding co-morbidities or age. In our study, when we compare the lymph nodes whether they are easily accessible or not (according to their location as deep or superficial), lymph nodes in CNB group were in deeper locations and hard to access. Despite the excessive number of tough lymph node locations in CNB group, 83.8% fully diagnostic ability of the CNB in our study was quite satisfying. The decision to proceed with either a CNB or SEB was totally up to the physicians’ discretion who was involved in the diagnostic process. Despite this being a retrospective study and there were not any specific pre-defined selection criteria to decide on the technique, like the age of the patient, location of the lymph node, and accessibility of the biopsy site, our results showed a statistically significant difference in terms of location of lymph nodes and age between the 2 group. As the results indicated in our cohort, patients in the CNB group have significantly deeper and un-accessible lymph node locations nevertheless they have 83.8% fully diagnostic ability. We can say that CNB is a viable option for deeper locations. The guidelines also support this kind of preference over SNB.^5^ Johl et al^12^ have reported that CNB comprised 15.7% of all biopsy samples in Kiel lymph node registry and was more preferred in elderly patients and to sample lymph nodes which are not easy to access. Despite these infrequent reports, Assaf et al^13^ have documented an increased frequency of CNB, comprising more than half of all samples, regardless of age, gender, or a clinical judgment suggesting a possible malignancy. Our results also indicated a statistically significant difference between the median ages of patients who underwent CNB or SEB (56.8 vs. 47.75 respectively, *P* = .003). Core needle biopsy was the most preferred technique among elderly patients as the first diagnostic tool because of frailty. According to our study population, preference rate of CNB was 44.1% in all cases. 

A 17-gauge coaxial needle with the 18-gauge core biopsy needle was used to obtain samples in our study. One equal size needle was used in all lymph nodes apart from the anatomical location. There are studies that favor to use different sizes of needles in deep or superficial samples,^14^ but we may conclude that using coaxial technique made it possible to obtain enough number of samples with a median of 11.5 (2-45) samples with just 1 puncture in our study. Even, there was no significant difference between number of samples obtained with the procedure and diagnostic ability in CNB group. 

The variation in diagnostic yield between lymphoma sub-types has previously been reported.^15^ There was significant difference between Hodgkin and non-Hodgkin lymphoma groups in 2 previous studies.^7^ Diagnostic yield of CNB in Hodgkin lymphoma was 50% in Burke et al^7^ study which evaluated 171 different patients presented with lymphoma from head and neck regions. Core needle biopsy was diagnostic in 30/38 (78.9%) of Hodgkin lymphoma patients in a retrospective cohort study including 114 lymphoma patients.^16^ This probably reflects the heterogeneity in histological background of lymph nodes in Hodgkin lymphoma and the chance of tissue sampling to detect critical features such as Reed–Sternberg cells might not be present in the core. In our study, CNB was 90.5% diagnostic in Hodgkin lymphoma patients. 

It is recognized that, despite the recent diagnostic advances, the relative lack of architectural assessment in core biopsies may result in difficulty in some cases, particularly in low-grade non-Hodgkin lymphomas and those with aberrant profiles.^17^ In terms of the diagnostic ability of CNB in follicular lymphoma, this entity is well described in literature; in our study, we found that CNB was 80% diagnostic in follicular lymphoma. Unfortunately, the total number of patients who were diagnosed with follicular lymphoma was only 10, and this might not exactly reflect the potency of CNB in the diagnosis of follicular lymphoma. DLBCL, another subgroup of non-Hodgkin lymphoma CNB, had a very high diagnostic ability with no need for further excisional biopsy.

Another important issue about CNB is false negativities or positivities. There are a few studies that evaluated predictive value of CNB in the literature. Data from the German cohort showed that 121 patients who underwent US-guided lymphadenopathy, 54 of them needed to be re-evaluated with second excisional biopsy because of the ambiguous cases.^18^ There were 2 false-negative and 2 false-positive patients in NHL group and 5 false-negative Hodgkin Lymphoma (HL cases in 76 lymphoma diagnosis. Also, CNB was sufficient in 65 of 76 lymphoma patients. Another study from Turkey has evaluated 291 patients who needed a second biopsy after following up to 6 months.^19^ Core needle biopsy was reported to be benign in 11 of 60 patients and 7 of 11 patients were misdiagnosed as having a lymphoma in this cohort. However, publications from single institutions like our study show a conclusive result for the diagnosis of lymphoma by CNB specimens in over 80%-90% of the cases. Negative CNB results should be considered with excessive precaution, and close follow-up and secondary biopsies are recommended according to the clinical aspects of the patient and radiological features of the affected lymph nodes. The lack of a comparison group consisting of the benign pathologies and their follow-up results is regarded as a limitation of our study. Besides, dedicated interventional radiologist to obtain the CNBs and a dedicated hemato-pathologist to report all the samples resemble the strength of our study.

This study has some limitations. One of our major limitation is retrospective nature of the data. Due to the retrospective nature of the data as we discussed above, there were not any specific predefined selection criteria to decide on between 2 techniques. It was all physician discretion. At this point, results obtained from the study described statistically significant differences in patient characteristics between CNB and SEB. However, these differences favor CNB group in terms of deeper location lymph nodes and advanced age, and theoretically could interpret as selection bias.

According to the results obtained in our study, we may conclude that CNB is a viable and comparable alternative to SEB, offering a less-invasive and less-expansive approach to diagnose lymphoma.

## Figures and Tables

**Table 1.** Patient Demographics, Biopsy Locations, and Diagnosis Among CNB and SEB Groups

	Core Needle Biopsy (n = 68)	Surgical Excisional Biopsy (n = 63)	*P*
**Gender, n (%)**			
Male	38 (55.9)	40 (63.5)	.375
Female	30 (44.1)	23 (36.5)	
**Age, median (range) **	56.8 (19-86)	47.6 (19-87)	.003*
**Number of samples obtained **	11.5 (2-45)	-	
**Location, n (%) **			
Head and neck	30 (44.1)	41 (65.1)	.001*
Chest	1 (1.5)	0 (0)	
Axilla	6 (8.8)	8 (12)	
Abdomen	19 (27.9)	2 (3.2)	
Inguinal	12 (17.6)	12 (19)	
**Location due to accessibility**			
Superficial	47 (69.1%)	60 (95.2%)	<.001*
Deep	21 (30.9%)	3 (4.8%)	
**Diagnosis**			
Hodgkin lymphoma	19 (27.9)	26 (41.3)	.315**
Non-Hodgkin lymphoma			
Diffuse large B cell lymphoma	11 (16.2)	7 (11.1)	
Peripheral T cell lymphoma	2 (2.9)	10 (15.9)	
High-grade B cell lymphoma	8 (11.8)	6 (9.5)	
Follicular lymphoma	10 (14.7)	9 (14.3)	
Marginal zone lymphoma	3 (4.4)	2 (3.2)	
Mantle cell lymphoma	6 (8.8)	0 (0)	
Atypical lymphoid proliferation	3 (4.4)	1 (1.6)	
Others	6 (8.8)	2 (3.2)	

*P < .05; **Hodgkin lymphoma and non-hodgkin lymphoma groups were compared.

CNB, core needle biopsy; SEB, surgical excisional biopsy.

**Table 2.** Diagnostic Sub-Categories of CNB Versus SEB

	Core Needle Biopsy (n = 68)	Surgical Excisional Biopsy (n = 63)	*P*
**Diagnosis (n,%)**			
Fully diagnostic	57 (83.8)	60 (95.2)	.028*, .938**
Partially diagnostic	9 (13.2)	1 (1.6)	
Inadequate	2 (2.9)	2 (3.2)	

*Three diagnostic groups were compared;**Fully and partial diagnostic yields were compared with inadequate yield.

CNB, core needle biopsy; SEB, surgical excisional biopsy.

**Table 3.** Need for a Second Biopsy

	Core Needle Biopsy (n = 68)	Surgical Excisional Biopsy (n = 63)	*P*
**Need for a second biopsy (n,%)**			
Yes	5 (7.4)	3 (4.8)	.720
No (total diagnostic yield)	63 (92.6)	60 (95.2)	
